# Recognising the neurological burden of onchocerciasis: the need to include onchocerciasis-associated epilepsy in onchocerciasis global health metrics

**DOI:** 10.1186/s40249-025-01297-6

**Published:** 2025-03-28

**Authors:** Luís-Jorge Amaral, Robert Colebunders

**Affiliations:** 1 Global Health Institute, University of Antwerp, Antwerp, Belgium; 2 Liverpool School of Tropical Medicine, Liverpool, UK

**Keywords:** Onchocerciasis, Epilepsy, Burden of disease, Mortality, Disability-adjusted life years, Prevention, Cost-effective, Onchocerciasis-associated epilepsy, Nodding syndrome

## Abstract

**Background:**

Historically, onchocerciasis has been recognised for its dermatological and ophthalmological manifestations, such as blindness. However, growing epidemiological evidence indicates that onchocerciasis is also associated with neurological complications, particularly onchocerciasis-associated epilepsy (OAE). These complications are not currently reflected in disease burden estimates and associated disability-adjusted life years (DALYs) for onchocerciasis.

**Main text:**

The most recent global burden of disease estimates for onchocerciasis in 2019 reported 1.23 million DALYs without accounting for OAE. Yet, a preliminary study suggested that 128,000 years of life lost to disability (YLD, a key component of DALYs) may be attributable to epilepsy in onchocerciasis-endemic areas of East and Central Africa. This figure, which would represent over 13% of the total onchocerciasis morbidity burden and 10% of the global epilepsy morbidity burden, is likely still an underestimation. Current disability weights for epilepsy YLD estimation may not fully capture the spectrum of OAE, which often involves nodding syndrome, developmental delays, motor disabilities, cognitive impairments and stigma. In regions where access to antiseizure medication treatment is sparse, poorly controlled seizures can exacerbate disability and lead to premature mortality. Targeted integrated strategies—combining onchocerciasis control measures with improved epilepsy care—could help address these critical gaps.

**Conclusions:**

Recognising OAE as part of the disease burden associated with onchocerciasis may encourage global health stakeholders to allocate resources for targeted interventions, thereby refining disease burden estimates, reducing disability, averting premature deaths and improving overall health outcomes.

**Graphical Abstract:**

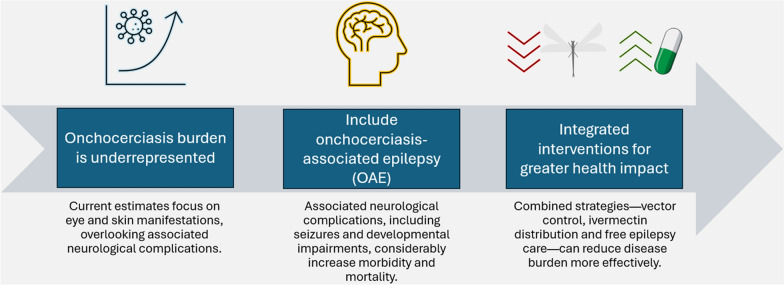

## Background

Using data from the 2021 Global Burden of Disease (GBD) study, Zhu and colleagues [1] assessed the prevalence and health burdens of vector-borne parasitic infectious diseases associated with poverty, revealed a statistically significant decrease in the overall burden of onchocerciasis since 1990. The estimated global age-standardised prevalence of onchocerciasis in 2021 was 246.2/100,000 population [95% uncertainty interval (UI): 222.7, 273.1], representing a 44.6% reduction (95% UI: − 46.6, − 42.6%) compared to 1990 [1]. Similarly, the corresponding estimated global age-standardised disability-adjusted life years (DALYs) of onchocerciasis was 15.8 per 100,000 population (95% UI: 9.4, 23.9), a 40.8% decrease (95% UI: − 45.0, − 37.1%) since 1990 [1]. DALYs, which combine years of life lost (YLLs) and years lived with disability (YLDs), incorporating disability weights to reflect severity. By merging both mortality and morbidity in one metric, DALYs capture the overall impact of a disease on populations. These encouraging results reflect effective onchocerciasis elimination efforts, based on community-directed treatment with ivermectin (CDTI) and, in some regions, complemented by vector control activities.

In 2021, the World Health Organization presented a 2021–2030 road map for neglected tropical diseases (NTDs), advocating an intensified global commitment against 20 NTDs, including the elimination of onchocerciasis [2]. Onchocerciasis is traditionally recognised for its dermatological and ophthalmological clinical manifestations, including irreversible blindness. However, consistent evidence strongly associates onchocerciasis with neurological complications with onset in children and adolescents, collectively termed onchocerciasis-associated epilepsy (OAE) [3–5].

Despite comprehensive epidemiological data supporting this association, OAE is not yet accounted for in existing burden-of-disease estimates and related DALYs of onchocerciasis [1]. Moreover, recent global health metrics on epilepsy have not incorporated data from high-transmission onchocerciasis-endemic regions, where OAE is found [6]. Since these estimates primarily rely on data from areas with low or no onchocerciasis transmission and apply standard epilepsy disability weights [6], they likely underestimate the burden specifically linked to OAE—including its severe clinical presentations such as nodding and Nakalanga syndromes. Therefore, OAE remains largely underrepresented in global health metrics, resulting in an incomplete estimation of its true burden.

While onchocerciasis has been eliminated as a public health problem in certain settings—particularly in parts of West Africa—other regions with limited healthcare infrastructure, ongoing conflict and weak elimination programmes continue to face intense *Onchocerca volvulus* transmission and a high prevalence of onchocerciasis-associated morbidity [3]. Some of these areas report a considerable prevalence (2.4–7.2%) of OAE [3]. A recent population-based study in two onchocerciasis-endemic health districts of the Central African Republic—where a prolonged civil war has hampered healthcare delivery and NTD control efforts—found that 9.1% (146/1600) of participants reported seizures [7].

## Main text

### The OAE disease burden

Onchocerciasis-associated epilepsy typically develops between the ages of 3 to 18 years, with clinical presentations that include a spectrum of seizures, such as generalised epilepsy, nodding syndrome (characterised by head-nodding seizures and cognitive impairment) and Nakalanga syndrome (marked by growth retardation and systemic deformities) [3]. In many onchocerciasis-endemic communities, these onchocerciasis-associated neurological manifestations exacerbate the disease burden through frequent seizures, learning difficulties and social stigma [3]. Such challenges not only disrupt education and limit employment opportunities but also perpetuate cycles of poverty. Additionally, people with epilepsy in onchocerciasis-endemic areas experience statistically significantly higher premature mortality rates compared to those with epilepsy in non-endemic regions, and are about five times more likely to die than their counterparts without epilepsy [8]. This excess mortality is primarily due to poorly controlled seizures, which can lead to status epilepticus, drowning, sudden unexpected death in epilepsy, burns and traumatic injuries [8].

One preliminary study estimated that approximately 128,000 YLDs are attributable to epilepsy in onchocerciasis-endemic regions of East and Central Africa [9]. This figure alone would account for over 13% of the total morbidity from onchocerciasis and 10% of the global epilepsy burden [9]. However, this estimate likelyunderestimates the true burden of OAE for three key reasons. First, disability weights used to calculate YLDs may not fully capture the severe manifestations of OAE [9], such as nodding syndrome, developmental delay and cognitive impairments. Second, limited access to and inconsistent adherence to antiseizure medication (ASM) in endemic areas exacerbate seizure frequency and disability, leading to worse health outcomes. Lastly, these factors also contribute to premature mortality, inflating the total DALYs via unaccounted YLLs.

Accurately estimating OAE-specific YLDs remains challenging, given that epilepsy has multiple potential causes even in high-transmission onchocerciasis settings. A practical approach to identifying OAE cases combines clinical and epidemiological criteria—such as the age of seizure onset between 3 and 18 years, residence in onchocerciasis-endemic areas and absence of other clear epilepsy aetiologies [3]. Although this definition cannot fully exclude other causes, it has proven effective for epidemiological surveys, especially in areas where neurocysticercosis is rare [4, 5]. Where feasible, confirmatory tests, such as serology for *O. volvulus* and basic neuroimaging, could improve diagnostic specificity [3].

To refine YLD estimates for OAE, functional impairment data (e.g., seizure frequency, ASM adherence and community-reported disability metrics) should be integrated with onchocerciasis prevalence indicators. Additionally, onchocerciasis statistical and modelling approaches are being developed to quantify the fraction of epilepsy linked *to O. volvulus* in regions where onchocerciasis transmission remains intense. By integrating epidemiological data with refined disability weights, mortality estimates and prevalence models, a more comprehensive assessment of the OAE burden can be achieved. These insights would not only strengthen DALY estimates but also inform cost-effective interventions aimed at reducing both *O. volvulus* transmission and its associated burden.

## The case for updating disease burden estimates

Incorporating the contribution of OAE into onchocerciasis burden estimates is essential for two overarching reasons. First, more accurate statistics could rally intensified global efforts toward onchocerciasis elimination. Second, robust disease metrics would justify the expansion of cost-effective interventions aimed at reducing OAE incidence, mortality and disability.

Underestimation of the GBD is not unique to OAE. It has been reported for other NTDs, including lymphatic filariasis, cutaneous leishmaniasis and Buruli ulcer [10]. Like OAE, these conditions contribute to substantial mental health burdens that, when considered, could significantly increase their disability weights [10]. Additionally, the impact on caregivers is often overlooked despite contributing considerably to the overall disease burden [10]. As these diseases are both preventable and treatable, increased investment in their control and elimination could yield major public health gains.

Interventions in Maridi County, South Sudan [4], and the Mahenge Area, Tanzania [5], illustrate how strengthening onchocerciasis elimination programmes can be followed by statistically significant declines in epilepsy incidence, including nodding syndrome. Both regions transitioned from annual to biannual CDTI in response to high OAE prevalence, accomplished without depending on external funding and leveraging ongoing donations of ivermectin by Merck & Co., Inc. Further low-cost, targeted strategies could be employed to boost ivermectin coverage. These could include distributing ivermectin in schools—given that children are most at risk for OAE—and possibly integrating this with praziquantel delivery in schistosomiasis co-endemic areas. Ivermectin could also be offered at hospital facilities to postpartum women one week after delivery—since they are excluded from treatment during pregnancy—and to individuals who missed the previous CDTI round [11]. In Maridi, a community-driven low-cost “Slash and Clear” vector control method was followed by an over 90% reduction in blackfly biting rates [4, 11]. Although initially reliant on small grants, this intervention is now sustained through local resource mobilisation.

Simultaneously, community-based epilepsy care programmes offering free ASMs can substantially reduce seizure frequency and epilepsy-related mortality. In Maridi, retrospective household data have shown decreased mortality following both strengthened onchocerciasis elimination efforts and the establishment of an epilepsy treatment centre providing free ASMs (*JN Siewe Fodjo & L-J Amaral, unpublished data*). Similar outcomes occurred in Mahenge, where enhanced onchocerciasis control and a community-based epilepsy programme were followed by a statistically significant decline in weekly seizure frequency, from a mean of 1.9 seizures at enrollment to 0.4 seizures at the last follow-up [12]. The majority of deaths in the Mahenge cohort were epilepsy-related (60%) and predominantly (88%) occurred among persons with epilepsy who did not fully adhere to the prescribed ASMs [12]. Annual costs of achieving seizure freedom for up to 70% of people with epilepsy can be as low as USD 5.2 per person when phenobarbital is delivered by trained primary healthcare workers, or USD 102.2 per person when carbamazepine is used [9].

These initiatives from South Sudan and Tanzania demonstrate the feasibility of integrated, cost-effective and sustainable measures that tackle both onchocerciasis transmission and its associated neurological sequelae and how these can achieve tangible benefits.

## Conclusion

Recognising OAE within existing onchocerciasis burden estimates has the potential to transform onchocerciasis elimination strategies. Recent robust epidemiological studies have demonstrated a strong association between onchocerciasis and epilepsy, underscoring the need to account for OAE in disease burden estimates. More reliable data on OAE prevalence, disability and mortality could catalyze global efforts, secure essential funding and foster the development of comprehensive morbidity management and disability prevention (MMDP) programmes. By acknowledging OAE as a key component of onchocerciasis-related burden, decision-makers can better allocate resources, implement integrated interventions (e.g., enhanced ivermectin distribution and epilepsy care) and ultimately curb the suffering imposed by this neglected tropical disease.

## Data Availability

Not applicable.

## Abbreviations

ASM: Antiseizure medication
CDTI: Community-directed treatment with ivermectin
DALYs: Disability-adjusted life years
DW: Disability weights
GBD: Global burden of disease
MMDP: Morbidity management and disability prevention
OAE: Onchocerciasis-associated epilepsy
YLD: Years lived with disability
YLL: Years of life lost
